# Safety of First-Line Nivolumab Plus Ipilimumab in Very Old (≥ 80 Years) Patients With Unresectable Malignant Pleural Mesothelioma: A Retrospective Single-Center Case Series

**DOI:** 10.7759/cureus.52714

**Published:** 2024-01-22

**Authors:** Takayuki Shimamoto, Yoshie Morimoto, Naohiro Nitta, Rie Yoshida, Nozomi Tani

**Affiliations:** 1 Department of Pulmonary Medicine, Kyoto Kuramaguchi Medical Center, Kyoto, JPN

**Keywords:** safety, case series, age, malignant pleural mesothelioma, nivolumab plus ipilimumab

## Abstract

Nivolumab plus ipilimumab as the first-line treatment results in superior survival outcomes in patients with malignant pleural mesothelioma (MPM). However, its safety in old (≥ 80 years) patients with MPM has not been elucidated yet. Three male patients with MPM, aged 80-90 years, were treated with nivolumab plus ipilimumab as the first-line treatment in our hospital. All of them discontinued the treatment due to adverse events. The overall survival from treatment initiation was 2.5, 3.5, and 4.0 months, respectively. Nivolumab plus ipilimumab should be used cautiously in very old patients with MPM.

## Introduction

Malignant pleural mesothelioma (MPM) is a highly aggressive disease with a five-year survival rate of approximately 10% [1]. Recently, the CheckMate 743 trial (first-line nivolumab plus ipilimumab in unresectable malignant pleural mesothelioma) demonstrated that first-line nivolumab plus ipilimumab is associated with superior overall survival (OS) compared to cisplatin plus pemetrexed in patients with MPM [2]. With the approval of immune checkpoint inhibitor (ICI) treatment for various types of cancer, they are being administered to patients with cancer from diverse health backgrounds [3].

MPM occurs in older individuals with a median age of 76 years [4]. In the CheckMate 743 trial, approximately 25% of patients were aged ≥ 75 years [2]. However, the efficacy and safety of nivolumab plus ipilimumab in patients with MPM aged ≥ 80 years (very old) have not been fully elucidated in a real-world setting.

Here, we report three cases of MPM in very old patients who were treated with nivolumab plus ipilimumab.

## Case presentation

Case 1

**Figure 1:**
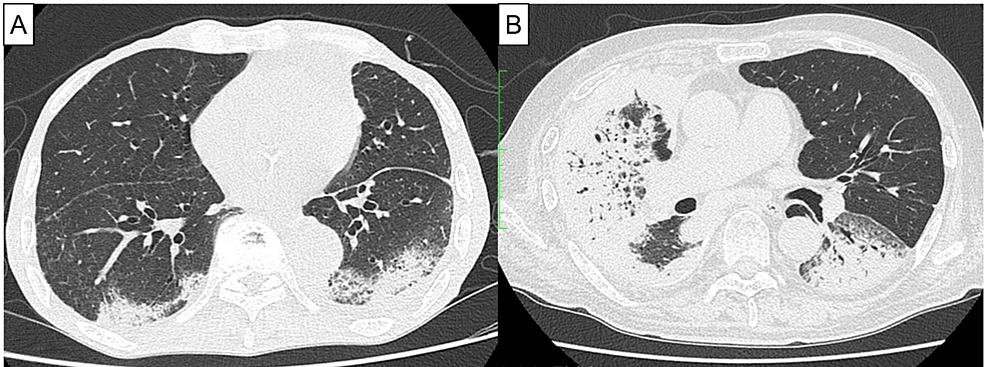
Chest CT at the onset of pneumonitis in two cases (A) A chest CT at the onset of pneumonitis in Case 1; (B) A chest CT at the onset of pneumonitis in Case 3

**Figure 2:**
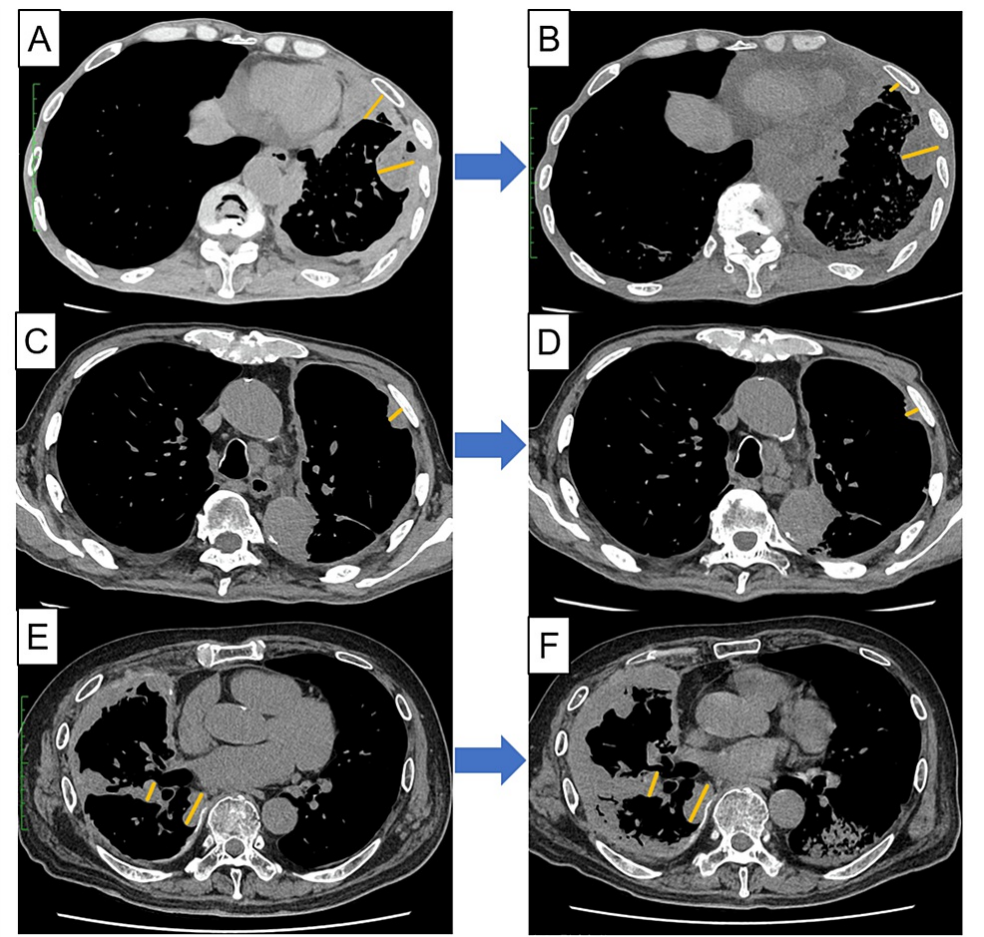
First CT evaluation during treatment with nivolumab plus ipilimumab The CT scan shows a size reduction in lung cancer in the first three cases. (A) A chest CT at the start of treatment in Case 1; (B) A chest CT after 2.5 months from treatment initiation; (C) A chest CT at the start of treatment in Case 2; (D) A chest CT after 1.8 months from treatment initiation (E); A chest CT at the start of treatment in Case 3; (F) A chest CT after 1.8 months from treatment initiation. Yellow lines indicate target lesions.

An 80-year-old man presented with left pleural thickening and effusion during a regular medical check-up. He previously worked in the building trade and had been exposed to asbestos. The patient’s performance status (PS) score was 2 points. MPM was diagnosed using video-assisted thoracoscopic surgery pleural biopsy; it was identified as the sarcomatoid variety. Nivolumab (360 mg every 3 weeks) plus ipilimumab (1 mg/kg every 6 weeks) was administered to him as the first-line treatment. Six weeks later, a chest computed tomography (CT) revealed pneumonitis. He had no symptoms, and the pneumonitis disappeared during follow-up. The second cycle was administered cautiously. However, the patient subsequently developed grade 2 pneumonitis, and the treatment was discontinued (Figure 1A). He had dyspnea at the onset of pneumonitis. Treatment response evaluation indicated stable disease (SD) (Figures 2A, 2B). Although the patient recovered from grade 2 pneumonitis, he died of aspiration 4.5 months after treatment initiation.

Case 2

A 90-year-old man presented with exertional dyspnea and weight loss. He was admitted to our hospital for close examination due to left pleural effusion and pleural thickening on chest CT. His PS score was 2. He worked at construction sites for eight years. CT-guided pleural biopsy confirmed the diagnosis of epithelial MPM. The patient received nivolumab (360 mg every 3 weeks) plus ipilimumab (1 mg/kg every 6 weeks) as the first-line treatment. Two months later, he presented with aspiration and died. The best objective response was SD (Figure 2C, 2D).

Case 3

An 80-year-old man presented to our hospital with fatigue. He had been exposed to asbestos during his plumbing work. His PS score was 1. CT-guided pleural biopsy revealed epithelial-type MPM. The patient received nivolumab (360 mg every 3 weeks) plus ipilimumab (1 mg/kg every 6 weeks) as the first-line treatment. After 1.8 months from treatment initiation, chest CT revealed pneumonitis and MPM progression (Figure 1B, 2E, 2F). He had a fever and dyspnea at the onset of pneumonitis. Nivolumab plus ipilimumab was discontinued due to adverse events (AEs) and disease progression. He was treated with 1 g methylprednisolone, and his pneumonitis improved. However, his general condition deteriorated, and he died four months after treatment initiation.

## Discussion

The study reported that three very old patients with MPM who were treated with nivolumab plus ipilimumab discontinued treatment owing to AEs. The backgrounds of all three cases are listed in Table 1. In Cases 1 and 3, the treatment was discontinued due to pneumonitis. In Case 2, it was discontinued due to grade 5 aspiration. Median PFS and OS from treatment initiation were 2.5 and 3.5 months, respectively, each being shorter than those in the pivotal study [2].

**Table 1:** Characteristics of the study patients AE, adverse event; BOR, best objective response; NE, not evaluable; OS, overall survival; PD, progressive disease; PFS, progression-free survival; PS, performance status; SD, stable disease; %FVC, percent forced vital capacity

Characteristics	Case 1	Case 2	Case 3
Age (years)	80	90	80
Sex	Male	Male	Male
Disease stage	IV	IIIB	IV
Tumor histology	Sarcomatoid	Epithelioid	Epithelioid
Smoking status (pack year)	25	0	22.5
Charlson comorbidity index	4	0	2
PS at the start of treatment	2	2	1
%FVC	70.2	41.3	NE
BOR of nivolumab plus ipilimumab	SD	SD	PD
Treatment discontinuation due to AEs	Pneumonitis	Aspiration	Pneumonitis
PFS from treatment initiation (months)	3.5	2.5	1.8
OS from treatment initiation (months)	3.5	2.5	4.0

CheckMate 743 included patients aged ≥ 80 years [2]. However, in real-world settings, only 30% of the patients are eligible for immunotherapy trials, and patients ineligible for clinical trials, who receive ICIs, have poorer OS than those who are eligible for clinical trials [5]. This highlighted the importance of evaluating the efficacy and safety of ICIs in old patients with cancer in real-world settings. Several studies have reported that Grades 3-5 AEs and treatment discontinuation occur at a higher rate in patients with non-small cell lung cancer (NSCLC) aged ≥ 75 years who received ICI-based therapy [6-8]. In a pooled analysis of first-line nivolumab plus ipilimumab in patients with NSCLC, the safety profile in patients aged ≥ 75 years was similar to that reported for the overall population; however, AE-related discontinuation rates were higher than those in patients aged < 75 years) [9]. Furthermore, in a cohort study evaluating the efficacy and safety of ICI monotherapy in patients with cancer aged ≥ 80 years, efficacy was suggested; however, discontinuation due to AEs was more common with increasing age [10]. Therefore, nivolumab plus ipilimumab should be chosen with caution for the treatment of very old patients with MPM.

 In our case series, two of the patients had a PS score of 2. In the CheckMate 743 trial, patients with a PS score of 2 were ineligible, and hence, were not evaluated. ICI monotherapy is moderately effective and tolerable, even in patients with a PS of 2 [3]. However, the efficacy and safety of nivolumab plus ipilimumab in patients with a PS score of 2 remain unclear. Old patients with cancer are more likely to have a poorer PS than younger patients, owing to their reduced ability to perform activities of daily living, history of multiple comorbidities, reduced organ function, and cognitive decline [3]. A PS score of 2 may have an impact on poor treatment outcomes.

## Conclusions

In conclusion, first-line nivolumab plus ipilimumab should be used with caution in very old patients with MPM. Further, large-scale clinical studies would be required to evaluate the clinical outcomes of very old patients with MPM who are treated with nivolumab plus ipilimumab.
